# Prevalence of hypothermia on admission to recovery room remains high despite a large use of forced-air warming devices: Findings of a non-randomized observational multicenter and pragmatic study on perioperative hypothermia prevalence in France

**DOI:** 10.1371/journal.pone.0226038

**Published:** 2019-12-23

**Authors:** Pascal Alfonsi, Samir Bekka, Philippe Aegerter, on behalf of the SFAR Research Network investigators

**Affiliations:** 1 Department of Anesthesiology, Groupe Hospitalier Paris Saint Joseph, Paris, France; 2 Clinical Research Unit Paris Ile-de-France Ouest (URCPO) and UMR 1168 UVSQ INSERM, Hôpital Ambroise Paré–AP-HP, Boulogne-Billancourt, France; Cleveland Clinic, UNITED STATES

## Abstract

**Background:**

Despite the availability of effective warming systems, the prevalence of hypothermia remains high in patients undergoing surgery. Occurrence of perioperative hypothermia may influence the rate of postoperative complications. Recommendations for the prevention of inadvertent perioperative hypothermia have been developed and are effective to reduce the frequency of perioperative hypothermia when professionals comply with. French Society of Anesthesiology (SFAR) decided to promote guidelines for the prevention of inadvertent hypothermia, and to conduct beforehand a pragmatic assessment of the prevalence of hypothermia in France. The hypothesis was that the rate of hypothermic patients (Tc<36°C) admitted to the RR remains high (around 50%), and that was the consequence of a warming device underutilization and/or was related to the type of health facilities.

**Methods:**

An observational, prospective and multi-centric study was conducted in France between October 2014 and May 2016 among patients over 45 years undergoing non-cardiac, non-outpatient surgery with anesthesia lasting >30 minutes in 52 centers. Patients undergoing pulmonary or proctologic surgery and those having non-invasive procedures performed under general anesthesia (for example, digestive endoscopy) were excluded from our study. Patients being operated under plexus anesthesia alone, surgeries involving hemorrhaging or infection, and patients presenting at least one organ failure were also excluded. The primary endpoint was the percentage of patients with a core temperature (Tc) <36°C on admission to the recovery room (RR).

**Results:**

Among 893 subjects (median age 66.9 years), prevalence of hypothermia on admission to the RR was 53.5%. At least one warming system was used for 90.4% of the patients. Identified risk factors for Tc<36°C included age≥70 years (OR = 1.41 [CI95%: 1.02–1.94]), duration of anesthesia from 1 to 2 hours (OR = 1.94 [CI95%: 1.04–3.64]) and a decrease in Tc of >0.5°C between anesthesia induction and surgical incision (OR = 1.82 [CI95%: 1.15–2.89]). Only a combination of pre-warming and intraoperative warming prevented a Tc<36°C (OR = 0.48 [CI95%: 0.24–0.96]).

**Conclusions:**

The prevalence of hypothermia among patients admitted to the RR remains high. Our results suggest that only the combination of pre-warming and intraoperative warming significantly decreases it.

## Introduction

General or neuraxial anesthesia causes a change in thermoregulation leading to the appearance of inadvertent hypothermia, which is defined as a core body temperature (Tc) below 36°C when corrective measures are not taken (active warming) [1]. Such perioperative hypothermia may be responsible for a range of adverse events on awakening from anesthesia and/or during the postoperative period [1,2]. For several decades, effective methods have been available to prevent perioperative hypothermia, based on the notion of heat transfer during anesthesia. Intraoperative active warming reduces the incidence of complications, especially infectious and cardio-vascular complications, and also reduces the number of perioperative blood transfusions [2–4].

In spite of data supporting active warming during surgery and the availability of effective warming systems, the prevalence of perioperative hypothermia remains extremely variable from one health facility to another, ranging from 4% to more than 70% [5,6]. One of the reasons for this considerable variability may be the under-utilization of warming systems. A survey on perioperative hypothermia conducted in 17 European countries has shown that active warming is used in only 38.5% of cases and that the perioperative temperature was monitored in only 19.4% of patients [7]. However, different studies report an elevated rate of hypothermic patients (Tc<36°C) admitted to the recovery room (RR) despite the use of warming systems [6,8–9].

Various recommendations for the prevention of inadvertent perioperative hypothermia promoting intraoperative cutaneous active warming have been developed [10–13]. When complying with the recommendations, it is possible to reduce the frequency of perioperative hypothermia [14,15] as well as the incidence of complications [15]. Regarding these positive results, the French society of anesthesiology (SFAR) decided to promote guidelines for the prevention of inadvertent hypothermia. In order to improve the effectiveness of these recommendations, SFAR decided to conduct beforehand a pragmatic assessment of the prevalence of hypothermia and the utilization (quantitative and qualitative) of warming systems. The objectives were to better understand the reasons for a failure to prevent perioperative hypothermia and to help health professionals improve their practices.

We hypothesized that the rate of hypothermic patients (Tc<36°C) admitted to the RR remains high (≈ 50%) in France, and that was the consequence of a warming device underutilization and/or was related to the type of health facilities.

## Materials and methods

After IRB (Comité de Protection des Personnes Paris Ile de France XI) approval and written informed consent, an observational, prospective and pragmatic and multicenter study was conducted in France. The aim of this observational study was to assess the prevalence of hypothermia and its consequences on patient’s stay in RR. If possible, an identification of risk factors is realized in order to better understand the reasons of failure to prevent perioperative hypothermia and to help health professionals to improve their practices. Inclusions began on October 8, 2014 and were closed on May 18, 2016.

### Eligibility criteria

Hypothermia prevention policy is affected by many factors. Also, it was not possible to conduct a survey considering that all types of surgical procedures or anesthetic techniques or health facilities ("center effect") were similar. In order to improve the accuracy of the results, a stratification has been performed on two factors that might influence the practice of hypothermia prevention: (1) the type of surgery and anesthesia and (2) the type and size of the health facility.

To enroll in the study, patients had to be 45 years or over and undergoing non-cardiac, non-outpatient surgery that would involve an anticipated duration of anesthesia of more than 30 minutes. The types of anesthesia included general anesthesia (combined or not with a loco-regional anesthesia) and neuraxial anesthesia (spinal or epidural). Patients undergoing pulmonary or proctologic surgery and those having non-invasive procedures performed under general anesthesia (for example, digestive endoscopy) were excluded from our study. We also excluded patients being operated on under plexus anesthesia alone, surgeries involving hemorrhaging or infection, and patients presenting at least one organ failure.

Because it was impossible to conduct an exhaustive study across the approximately 1,500 healthcare facilities in France that perform surgery, we worked on a random sample. We performed a stratified self-weighted two-stage sampling, with stratification considering i) hospital funding ((university hospital, general hospital and private clinic) and ii) annual volume of surgical procedures, as given by national DRG database (more or less 1000 procedures a year, settings with less than 500 annual procedures being excluded). In each stratum, the number of selected primary units (hospitals) was proportional to the number of secondary units (procedures) contained in the stratum and hospitals were drawn with unequal probabilities, proportional to the number of annual procedures. At the second level, in each selected hospital, a constant number of interventions were studied. As all the different centers that took part in the study did so on a voluntary basis, a complementary waiting list was calculated. At last, the resulting distribution was: 21% university hospitals, 25% general hospitals 111 and 54% private clinics. The duration of the study in each center was limited to two weeks, with the goal of recruiting 20 patients per center. This local patient population guaranteed the study’s credibility locally and made it possible to provide feedback. In addition, this number of patients corresponded to the volume of activity that one center, even a small one, can generate in two weeks.

### Local conditions and conduct of the study

An investigator was designated in each center (SI Appendix 1). The role of each investigator was to obtain patient’s consent and to help the clinical research assistant (CRA) to collect data. In order to respect the clinical practices of each hospital or clinic, the study was conducted in such a way as to not change the teams’ routine practices with patients in the operating room and in the RR. In particular, it was important for temperature monitoring and the use of warming systems for the duration of the anesthesia to be carried out “as usual.” In practical terms, this meant that the anesthetists were the sole judges of the need to use perioperative temperature monitoring and/or a warming system. To limit any potential bias, CRAs were specially trained and sent to each participating study center. For each patient, the CRA collected all the data and set up the SpotOn^™^ (3M, France) temperature monitoring system. The temperature displayed on the monitor was hidden to the investigator.

### Data collection

The collection will be carried out by a Clinical Research Assistant (CRA) with the help of the local investigator in each facility. The patient had a unique reference for this study anonymized in the format of “center code/inclusion number/initials Last Name-First Name”. Patient identities remained in the investigator site (including the register of patients).

Warming techniques, surgical and anesthetic data were entered on paper during the procedure as well as temperatures before anesthesia and on arrival in SSPI. The CRA entered later the collected data into a database.

### Core temperature (Tc) monitoring

To avoid measurement bias due to variations related to the technology and/or the site of measurement [16], the same thermometer (SpotOn^™^, 3M, France) was used in all patients. This non-invasive thermometer reflects Tc measured in the cerebral tissue, and provides continuous data. This technology is comparable to others for Tc monitoring [17–19].

Temperatures displayed on the SpotOn^™^ monitor were recorded at different points in time: (1) just before induction of anesthesia, (2) at the time of surgical incision, (3) at the end of surgery, (4) when leaving the operating room (OR), (5) when arriving at the RR, and (6) when leaving the RR. The use and type of warming system was also recorded. If a cutaneous warming system was used, times of start and stop were recorded.

### Other data

Other data included type of care facility, patient’s ASA classification, administration of an anxiolytic premedication, type of surgery, type of anesthesia, and medications administered during the procedure and perioperative temperature monitoring. For each intervention, the risk of bleeding was assessed based on predefined criteria [20]. High bleeding-risk procedures included intra-abdominal, major orthopedic and urological surgeries; peripheral artery revascularization procedures; and surgeries that lasted for more than one hour.

The use and type of warming system (cutaneous, intravenous fluids or irrigation fluids) was also recorded. If a cutaneous warming system was used, times of start and stop were recorded. If a local temperature monitoring was used, the measurement site (esophagus, bladder, etc.) was noted. During the postoperative period, the 4 following parameters were recorded: Tc at exit for RR, continuation of respiratory support in patients operated under general anesthesia, need for active warming, and amount of time spent in the RR.

### Endpoints

The primary endpoint was hypothermia on admission to the RR. In line with published recommendations about perioperative hypothermia prevention [8,9], patients with a Tc<36°C on admission to the RR were considered to be hypothermic.

Secondary endpoints were the factors influencing Tc before the start of anesthesia, and the initial drop in Tc between the induction of anesthesia and the surgical incision (DeltaTc_*init*_). The consequences of hypothermia on conditions in the RR were assessed through the 4 collected parameters.

### Statistical analysis

Taking as our hypothesis that the prevalence of hypothermia (Tc<36°C) is 50% at admission to the RR, and considering a clustering effect, our initial population was set at 1,600 subjects to obtain precision of ± 3% with an error risk of 5%. Since we expected a ratio of 25% of unusable patient records, the population size was initially adjusted to 2,000 patients in 100 centers. Following enrolment of ≈10% of the population, an intermediate analysis was programmed to check the quality of data collection and the percentage of hypothermic patients on RR admission. Based on the recruitment of 203 patients, the prevalence of hypothermia (Tc< 36°C) reached 60%. Finally, a study population of 800 patients was judged sufficient for a representative survey. Since we expected a ratio of 25% of unusable patient records, the population size was adjusted to 1,000 patients.

Data are expressed in percentage for qualitative or ordinal variables, and in median and interquartile range (IQR) [25%-75%] for quantitative variables. All statistical analyses were performed using R software (R Development Core Team, 2012. https://www.r-project.org/). Comparisons of the distributions of the qualitative variables used the Chi-Square test or Fisher’s exact test (as needed). Comparisons of continuous quantitative or ordinal data were performed with a non-parametric Kruskal-Wallis test.

Considering cutaneous warming modalities, patients were put into one of three groups: (1) Absent or ineffective warming group *(No-warming)* included patients who did not received any active warming, those for whom the duration of warming was at least 60 minutes less than the duration of anesthesia, and those for whom a blanket was not connected to a forced-air warmer; (2) Pre-warming and intraoperative warming group *(Pre&IO-warming)* included all patients for whom active warming began at least 10 minutes before induction of anesthesia [21] and was continued throughout anesthesia; and (3) Intraoperative warming group *(IO-warming)* included patients for whom active warming was started after induction of anesthesia and patients for whom intraoperative warming was stopped at least 10 minutes before the end of anesthesia.

For the hypothermia on RR admission, an attempt was made to identify independent risk and prevention factors. For the multivariate analysis, we used generalized linear mixed models and the logit function as a link function to obtain the adjusted Odds Ratio (OR) of the risk and prevention factors (with 95% confidence interval). The list of explanatory variables introduced into the model were based on the results of univariate analysis (p<0.05) as well as factors identified in the literature as playing a role in the occurrence of perioperative hypothermia [22–24]. Thus, were considered: age, sex, BMI and ASA score of the patient together with the main comorbidities and analgesics known to have an effect on thermoregulation including tramadol, morphine or nefopam. At last, characteristics of anesthesia and intervention were analyzed. For consequences of hypothermia, a multivariate analysis was conducted in order to assess if hypothermia on RR admission independently impacted conditions of stay in the RR.

## Results

**Fig 1:**
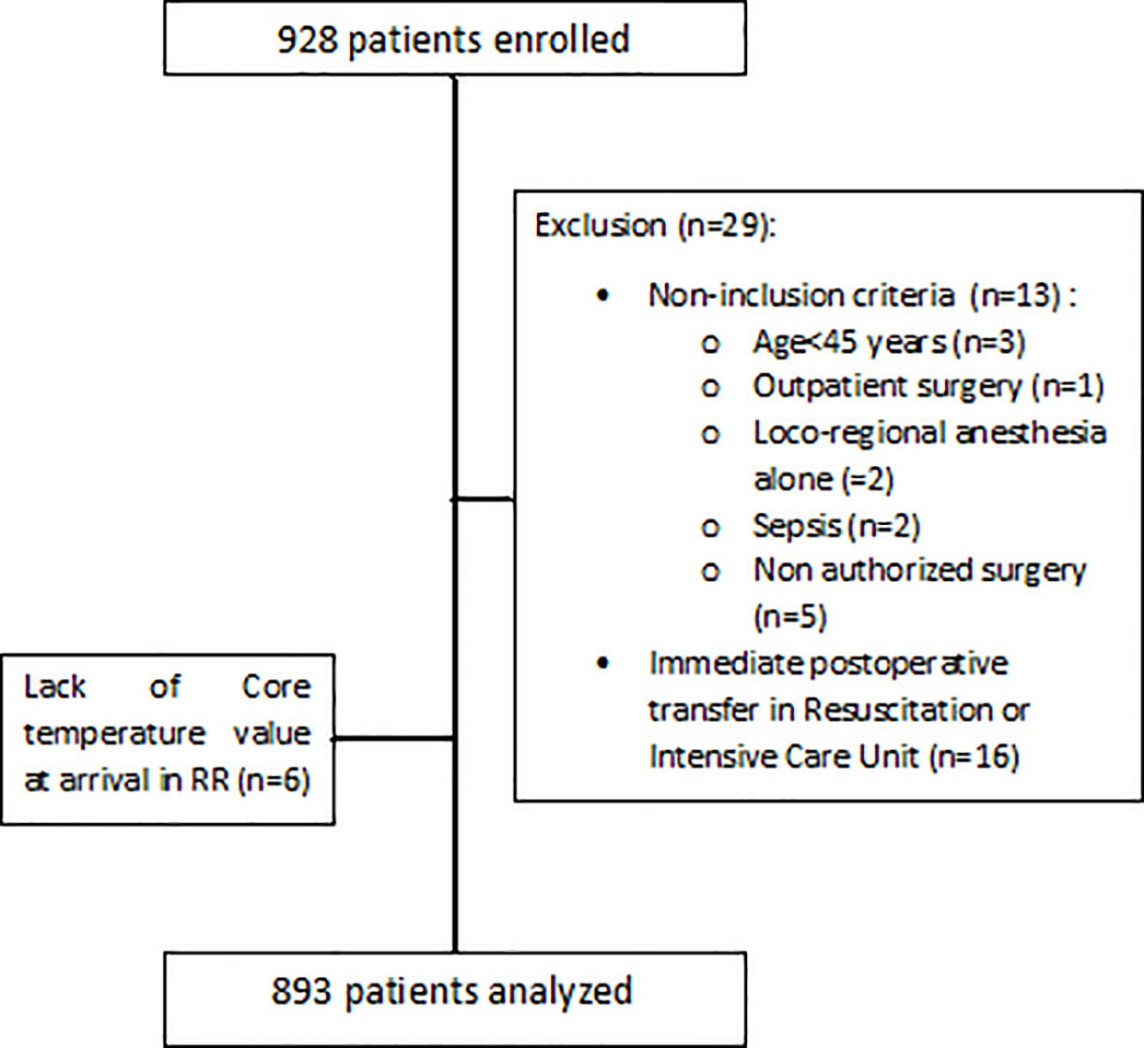
Flow chart.

Between October 2014 and May 2016, 928 patients were enrolled in 52 centers in France, or 17 (range: 5–27) patients by center. Before analysis of the data, 29 patients (3.1%) were excluded because they presented one of the non-inclusion criteria (n = 14) or because they were transferred directly to the intensive care unit (n = 15) (Fig 1). An additional 6 patients (0.6%) were excluded from the analysis because Tc on RR admission was missing. Finally, 893 (96.3%) patients were analyzed: 252 patients (28.2%) undergoing surgery in 15 (28.8%) university hospitals; 222 patients (24.9%) undergoing surgery in 14 (26.9%) public hospitals; and 419 patients (46.9%) undergoing surgery in 23 (44.2%) private clinics. Median age of the patients (50.4% female) was 66.9 years [IQR: 58–75.3] and 32.6% of them had an ASA score 3 or 4. The duration of anesthesia was 131 min [IQR: 90–200] and the length of stay in RR was 1.9 hours [IQR: 1.3–2.6]. Characteristics of patients and surgery and anesthesia are summarized in Tables 1 and 2. A temperature monitoring was used for 28.1% of the patients in the OR and for 42.7% in the RR. The proportion of patients who had temperature monitoring during surgery differed significantly according to the number of warming systems used: 2.3% with no warming system, 26.3% with one warming system (fluid or cutaneous) and 66.3% when both types of warming system were used (p<0.001). Cutaneous and/or fluid active warming was provided for 90.4% of the patients. A cutaneous warming system was used with all actively warmed patients except for one.

**Table 1:** Patient and surgery characteristics (n = 893).

Characteristic	n (%)
**Age ≥ 70 years**	354 (39.6%)
**Female gender**	450 (50.4%)
**Body mass index (kg/m^2^)**	
< 25	305 (34.5%)
Between 25 and 29.9	351 (39.7%)
≥ 30	228 (25.8%)
**ASA Physical status**	
1 or 2	601 (67.3%)
3 or 4	292 (32.6%)
**Co-morbidities**	
Hypertension	451 (45%)
Dyslipidemia	154 (25.6%)
Diabetes requiring medication	147 (16.6%)
History of smoking within 2 yrs. before surgery	123 (14%)
Peripheral arterial disease	50 (5.7%)
Stroke or transient ischemic attack	43 (4.9%)
Coronary artery disease	41 (4.6%)
Congestive heart failure	38 (4.3%)
Preoperative serum creatinine > 2.0mg/dL or. >175 μmol/L	15 (1.7%)
**Health facilities**	
Private clinics	23 (46.9%)
General hospital	14 (24.9%)
University hospital	15 (28.2%)
**Type of surgery**	
Orthopedic	308 (34.5%)
General	244 (27.3%)
Urologic	158 (17.7%)
Gynecologic	111 (12.4%)
Vascular	65 (7.3%)
Other	7 (0.7%)
**High-risk bleeding surgery**	88.1%

Abbreviation: IO = Intraoperative.

**Table 2:** Anesthetic characteristics (n = 893).

Characteristic	%
**Anxiolytic premedication**^#^	
None	42.9%
Benzodiazepine	28.7%
Hydroxyzine	18.6%
Gabapentanoids	14.0%
**Duration of anesthesia**	
>30 min and ≤60 min	7.7%
>60 min and ≤120 min	36.3%
>120 min and ≤180 min	25.1%
>180 min	31.0%
**Type of anesthesia**	
General anesthesia	71.1%
Combined general anesthesia and plexus analgesia	15.2%
Neuraxial anesthesia	9.4%
Combined general and neuraxial anesthesia	5.4%
**IO use of vasoactive drugs**	53.2%
**IO administration of postoperative analgesics**^#^	
At least one analgesic	89.6%
Paracetamol	80.4%
Nefopam	54%
Tramadol	31%
Non-steroidal anti-inflammatory Drug	27%
Morphine	24.6%
Other	0.6%

Abbreviation: IO = Intraoperative.

^#^ Total is greater than 100% because one patient may have received 2 or more medications.

**Fig 2:**
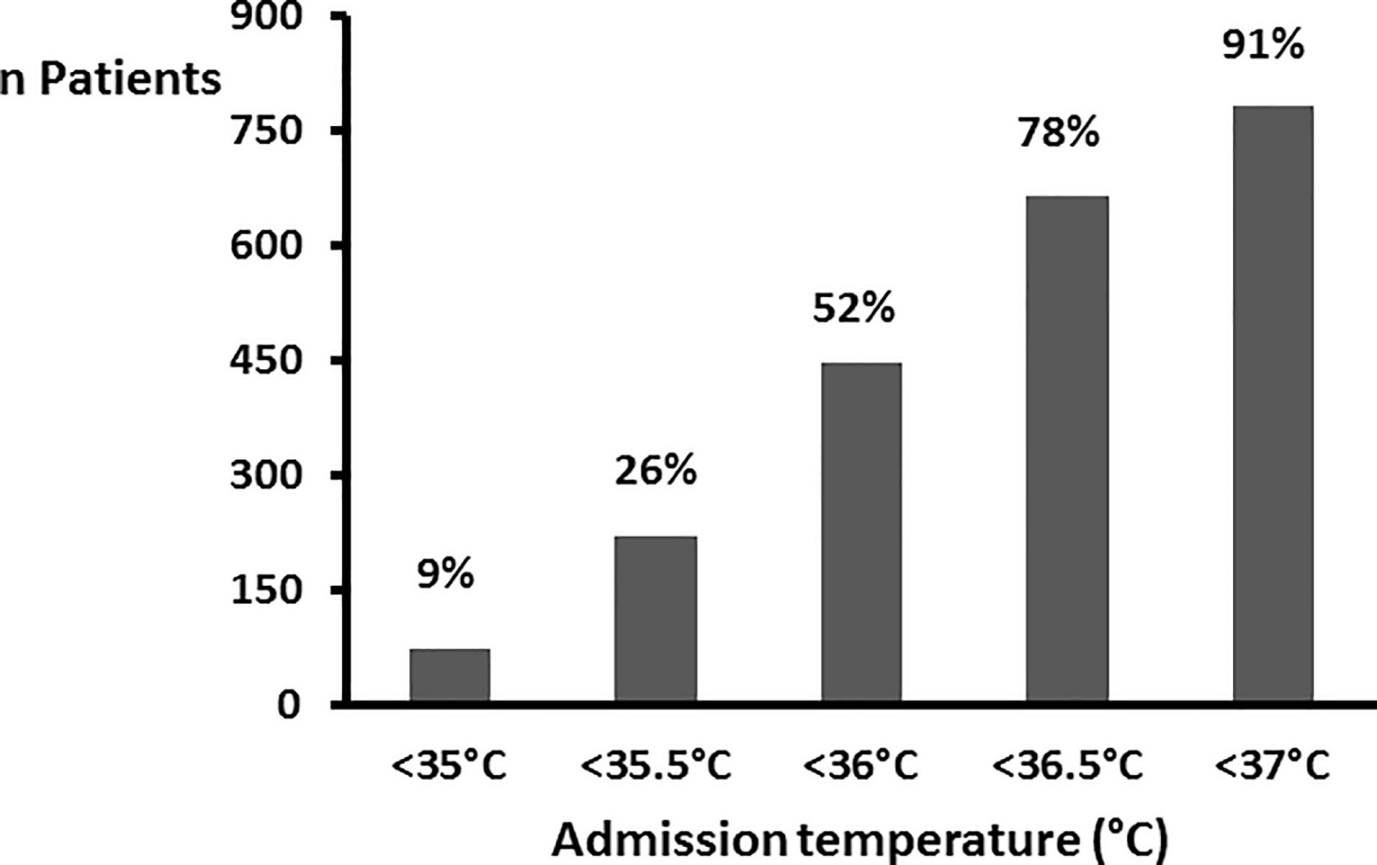
Breakdown of patients based on Tc when arriving at the recovery room. In a study published in 1981, Vaughan et al. [25] observed that 60% of patients arriving at the RR had Tc<36°C. The decrease in the prevalence of hypothermia is minimal (-8%), despite the invention and widespread adoption of pulsed-air warmers.

On admission to the RR, 53.5% of patients were hypothermic (Tc<36°C) with half of them (26%) with a Tc ≤ 35.5°C (Fig 2). By contrast, 20.3% of the patients had a Tc ≥ 36.5°C on RR admission. Prevalence of hypothermia was respectively 44.1% when anesthesia lasted between 31 and 60 minutes, 58.3% between 1 and 2 hours, 54.1% between 2 and 3 hours, and 49.6% for more than 3 hours (p = 0.07). A Tc decrease above 0.5°C between induction of anesthesia and admission at RR was observed in 57.2% of the patients. Among these patients, 28.3% had a Tc≥ 36°C on admission in the RR.

Hypothermia on arrival in RR was a factor for active warming in RR (OR = 3.53 [CI95%: 2.28–5.47]; p<0.001) and hypothermia at leaving the RR (OR = 5.85 [CI95%: 3.93–8.7]; p<0.001). When the patients were hypothermic, the length of stay in RR was not prolonged (ß = 0.28 [CI95%: -0.04–0.59]; p = 0.087) and the requirement of a mechanical ventilation in patients operated under general anesthesia was not increased (OR = 1.37 [CI95%: 0.92–2.06]; p = 0.1).

Before induction of anesthesia, the Tc was 36.6°C [IQR: 36.2–36.9] (Table 3). Before induction of anesthesia, 69.3% presented a Tc between 36°C and 37°C, 16.2% had a Tc ≤36°C, and 24.5% had a Tc ≥37°C. At least 10 minutes of pre-warming before the beginning of anesthesia was applied in 28.6% of the patients. Between the induction of anesthesia and the surgical incision, Tc decreased to 36.2°C [IQR75%: 35.8–36.5]. The initial Tc decrease (DeltaTc_*INIT*_) was not the same for all patients: over 0.5°C in 33.8% of the cases, and between 0 and 0.5°C in 45.8%. Tc was stable or slightly increased in the remaining patients (20.4%). After the surgical incision, perioperative Tc continued to decrease, reaching 36.1°C [IQR75%: 35.6–36.5] by the end of surgery and 36.0°C [IQR75%: 35.5–36.5] when the patient left the operating room. On admission to the RR, the Tc was 35.9°C [IQR75%: 35.4–36.3] (Table 3). Tc decrease between the beginning of anesthesia and the arrival at RR was 0.7°C [IQR75%: 0.2–1.1] at a rate of 0.3°C/hr [IQR75%: 0.2–1.1]. The major part of the drop occurred between the induction of anesthesia and the surgical incision: 0.4°C [IQR75%: 0.1–0.7] at a rate of 0.8°C/hr [IQR75%: 0.2–1.4].

**Table 3:** Core temperatures, temperature monitoring and IO active warming system use.

	Median [IQR75%]—or %
**Core temperatures—°C**	
Before anesthesia	36.6 [36.2–36.9]
At surgical incision	36.2 [35.8–36.5]
End of surgery	36.1 [35.6–36.5]
Exit of OR	36.0 [35.5–36.5]
Admission at RR	35.9 [35.4–36.3]
Exit of RR	36.2 [35.8–36.6]
**Delta Tc**_***init***_**—% patients**	
≥0°C	20.5%
Between -0.5 and 0°C	45.8%
≤ -0.5°C	33.8%
**IO temperature monitoring**	28.1%
*Esophageal*	*70*.*1%*
*Cutaneous*	*11*.*2%*
*Other (bladder*, *infrared tympanic*, *nasopharyngeal*, *etc*.*)*	*18*.*8%*
**IO Tc monitoring according to health facilities**	
University hospitals	57.1%
General hospitals	19.8%
Private clinics	15%
**Tc monitoring in RR according to health facilities**	
University hospitals	51.6%
General hospitals	50.9%
Private clinics	32.9%
**Use of OR warming devices**	
None	7.8%
Forced-air warmer	90.3%
(*including without a blanket*)	(*2*.*9%*)
i.v. fluid warmer	9.9%
Irrigation fluid warmer	0.7%
**Modalities of IO warming devices**	
Cutaneous warming alone	88.5%
Cutaneous warming and i.v. fluids	11.3%
Fluids alone	0.2%
IO active cutaneous warming modalities*	
*No-Warming*	22.7%
*IO-Warming*	66.4%
*Pre&IO-Warming*	10.9%

Abbreviations: Tc: core temperature. RR: Recovery Room. IO: Intraoperative. OR: Operating Room. DeltaTc_*INIT*_ (°C) corresponds to the core temperature difference between surgical incision and anesthesia induction.

*For the breakdown of patients in each sub-group, see “Methods” section.

**Fig 3:**
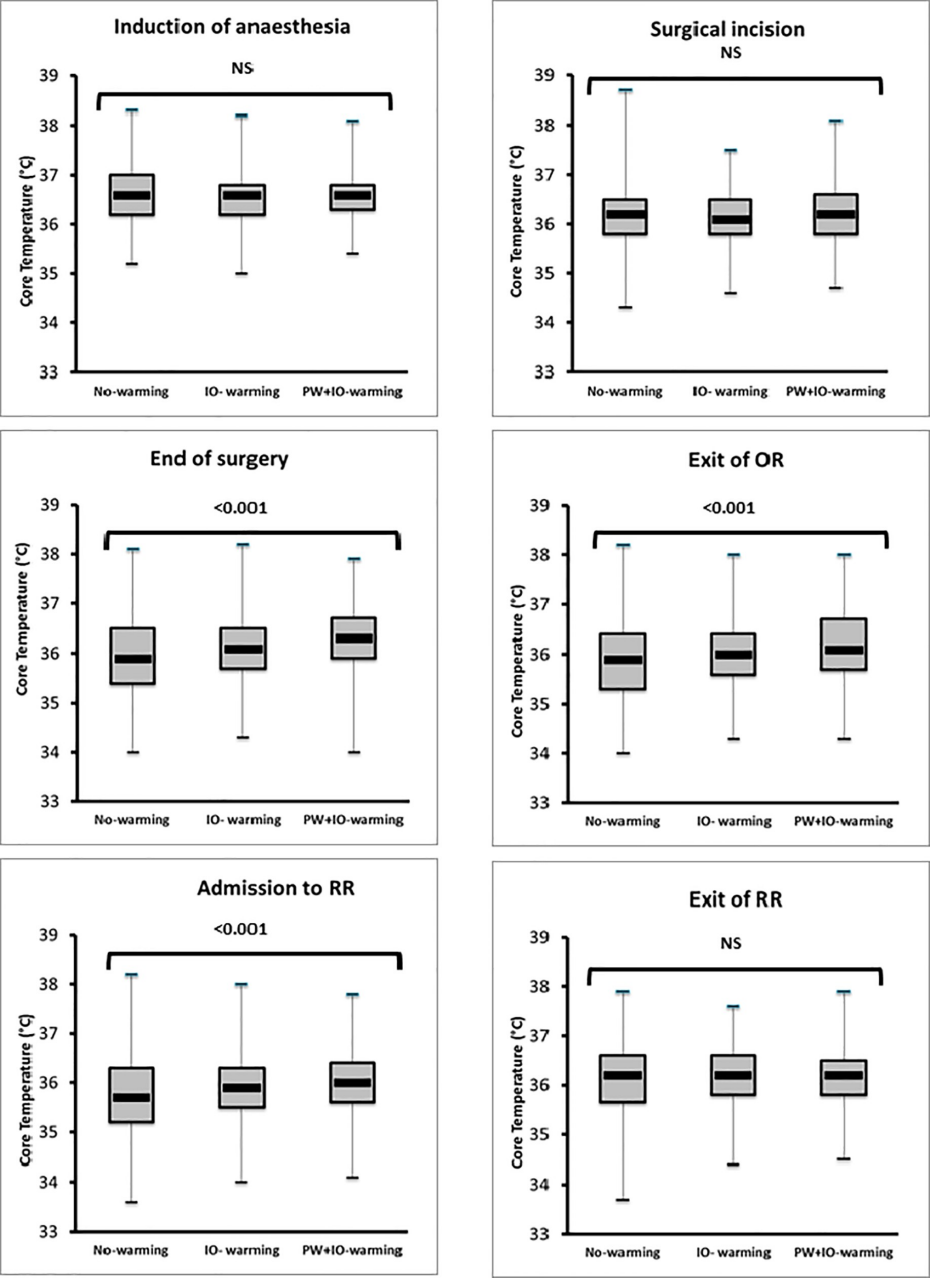
Box and whisker plots of core temperature according to intraoperative warming modalities. The horizontal line within the box indicates the median, boundaries of the box indicate the 25th- and 75th -percentile, and the whiskers indicate the highest and lowest values of the results. Core temperatures were not significantly different from the beginning of the anesthesia to the surgical incision time. They were significantly different at the end of the surgery and at arrival in the RR. They were again no statistically different when the patients left the RR.

The breakdown of patients in each warming modality is presented in Table 3. Among the 66.4% of patients into the *IO-warming* group, only 23% were actively warmed throughout the anesthesia (i.e. start of active warming no more than 10 minutes after the beginning of anesthesia and stop less than 10 minutes before the end). Intraoperative cutaneous warming modalities significantly influenced core temperatures (Fig 3). Tc were significantly different at the end of the surgery (p<0.001): *No-warming* (35.9°C [IQR: 35.4–36.5]), *IO-warming* (36.1°C [IQR: 35.7–36.5]), and *Pre&IO-warming* (36.3°C [IQR: 35.9–36.7]). The values were also significantly different at arrival in the RR (p<0.01): *No-warming* (35.7°C [IQR: 35.2–36.3]), *IO-warming* (35.9°C [IQR: 35.5–36.3]), and *Pre&IO-warming* (36.0°C [IQR: 35.6–36.4]).

Temperature monitoring during surgery differed significantly according to the number of warming systems used: 2.3% with no warming system, 26.3% with one device and 66.3% with two devices (p<0.001).

During the time in the RR, Tc gradually rose to reach 36.2°C [IQR75%: 35.8–36.6] before the patient was transferred to another department. On leaving the RR, 33.6% of the patients continued to present a Tc <36°C. Among this group, 50% were hypothermic when they arrived in the RR, while 15.2% of the patients were not (p<0.001).

Results of univariate and multivariate analysis are summarized in Table 4. Risk factors for hypothermia at arrival in RR were: age ≥70 years (OR = 1.38 [CI95%: 1–1.92]), duration of anesthesia between 1 and 2 hours (OR = 1.96 [CI95%: 1.03–3.74]) and a DeltaTc_*init*_*>0*.*5°C* (OR = 2.2 [CI95%: 1.36–3.56]). Among the warming modalities, only the combination of pre-warming and IO warming prevented Tc<36°C on admission to the RR (OR = 0.43 [CI95%: 0.21–0.88]).

**Table 4:** Predicting factors of hypothermia on recovery room admission.

	n Patients	Tc<36°C on RR admission	Univariate analysis	Multivariate analysis
			*p*	OR [CI95%]
**Age ≥ 70 yrs.**	354	59.6%	0.004	1.41 [1.02–1.94]
**Gender female**	450	54.4%	>0.5	0.99 [0.72–1.34]
**Health facilities**			0.003	
Private Clinic	419	59.4%		Ref
University Hospital	252	50%		0.7 [0.42–1.17]
General Hospital	222	46.4%		0.63 [0.38–1.03]
**Premedication with gabapentanoids**	125	62.4%	0.041	1.45 [0.86–2.43]
**Duration of anesthesia**			0.07	
>30 min and ≤60 min	68	44.1%		Ref
>60 min and ≤120 min	321	58.3%		1.94 [1.04–3.64]
>120 min and ≤180 min	222	54.1%		1.66 [0.85–3.25]
>180 min	274	49.6%		1.43 [0.71–2.9]
**IO use of i.v. lidocaine**	187	46.5%	0.038	0.68 [0.32–1.45]
**Use of IO active warming devices**			0.014	
None	86	62.8%		Ref
Cutaneous or fluids	715	54%		0.75 [0.35–1.61]
Cutaneous and fluids	92	41.3%		0.55 [0.22–1.4]
IO cutaneous warming modalities*			0.025	
No-warming	201	60.7%		Ref
IO-warming	587	52.5%		0.78 [0.47–1.31]
Pre&IO-warming	96	44.8%		0.48 [0.24–0.96]
**IO temperature monitoring**	251	48.2%	0.055	0.94 [0.62–1.42]
**Tc before induction of anesthesia**			<0.001	
≤36°C	131	82.4%		Ref
>36°C	738	48.1%		0.16 [0.09–0.27]
**DeltaTc**_***init***_ **(°C)**			0.004	
≥0°C	176	54.5%		Ref
Between -0.5 and 0°C	393	47.6%		0.81 [0.53–1.24]
≤ -0.5°C	290	60.3%		1.82 [1.15–2.89]

Abbreviations: Tc = core temperature; IO = Intraoperative; OR = Odds Ratio. DeltaTc_*init*_ corresponds to the core temperature difference between surgical incision and anesthesia induction.

*For the breakdown of patients in each sub-group, see “Methods” section.

The predisposing factors of a Tc ≤ 36°C before the start of anesthesia were: age ≥70 years (OR = 1.48 [CI95%: 1.08–2.04]; p<0.001) and premedication with a benzodiazepine (OR = 1.42 [CI95%: 1–2.04]; p = 0.006). Pre-warming for at least 10 minutes did not influence the Tc immediately before the start of anesthesia (OR = 1.21 [CI95%: 0.8–1.83]; p = 0.36).

The prevalence of a DeltaTc_*init*_*>0*.*5°C* was lower among patients over age 70 (OR = 0.68 [CI95%: 0.51–0.9]; p = 0.008) or when anxiolytic premedication with a benzodiazepine was prescribed (OR = 0.7 [CI95%: 0.52–0.95]; p = 0.024). At least 10 minutes of prewarming did not influence the DeltaTc_*init*_*>0*.*5°C* (OR = 0.88 [CI95%: 0.64–1.22]; p = 0.45).

## Discussion

More than 35 years ago, Vaughan et al. [25] reported that 60% of patients had core temperatures of <36°C on admission to the RR. With the development of effective warming devices (i.e., pulsed-air warmers) since the early 1990s, we should have observed a decrease in this rate. Our study highlights that today in France we have not witnessed any real progress: 53.5% of patients who are given anesthesia for at least 30 minutes are hypothermic (Tc<36°C) when they are admitted to the RR. What’s more, the core temperature of 1/3 of patients remains below 36°C when they return to the surgical ward.

A survey published in 2007 showed that fewer than 2 out of 5 patients received active warming during surgery in Europe [7]. Nowadays, an active warming system is used for 90% of patients at some point during anesthesia and an underutilization of equipment is unlikely an explanation of the high observed rate of hypothermic patients in our study. Burns et al. [5] report that with a utilization rate of 96%, only 4% of patients are hypothermic on admission to the RR. When local guidelines are applied in a hospital, only 10% of the patients have a Tc ≤36°C on admission to the RR [15]. However, various authors [6,8–9] have reported levels of hypothermia similar to our findings despite the use of warming devices. Beyond the use of warming systems, it is *how* they are used that is important. Amount of heat transferred into the body with a pulsed-air warmer depends on the time ratio between anesthesia duration and active warming duration. Only one-third of the patients warmed during the intraoperative period can be considered to have received heat long enough. This point could explain why an IO cutaneous warming alone was not significant for preventing hypothermia, in comparison with other reports [5, 15].

Combined pre-warming and intraoperative warming reduces significantly the rate of hypothermic patients at arrival in the RR. However, the percent of hypothermic patients (44.6%) is far more than expected. Interestingly, we observed a 0.2°C fall of the Tc between the end of the surgery and the arrival at the RR whatever the method used or not for warming the patient. Even in the combined pre-warming and IO warming sub-group, the percent of patients with a Tc <36°C increased from 20–25% to 50% during the transfer from the operating room to the RR. That means that this period is at risk of cooling for the patient because, in the meantime, active warming is stopped and most of the patients have sub-anesthetic concentrations of drugs and/or have received medications (e.g. analgesics) with an inhibitory thermoregulatory effect [1].

From our data, the decrease in core temperature may be roughly divided into 2 phases: from the start of anesthesia to the surgical incision and from the beginning of surgery to the RR. Core temperature does not decrease evenly over these 2 periods. During the initial phase a rapid drop-in Tc corresponding to almost 60% of the total decrease occurred at a rate of 0.8°C/hr. During the following phase, the rate of cooling is slower. One third of the patients lose more than 0.5°C during the initial phase. This drop doubles the risk of having a Tc<36°C on admission to the RR, suggesting that initial decrease in core temperature cannot be easily compensated for during surgery, regardless of the duration and the use of cutaneous warming. In this context, reducing the extent of the initial decrease is an objective to pursue if we want to successfully reduce the incidence of hypothermia in the RR. Two mechanisms are responsible for perioperative hypothermia: internal redistribution of heat and heat loss [26]. Among the 2 mechanisms leading to hypothermia during the anesthesia, the internal redistribution is the primary mechanism responsible for the drop-in core temperature between anesthesia induction and surgical incision. To avoid the initial drop in Tc, some have suggested warming the peripheral compartment before induction of anesthesia (pre-warming) [1]. Moreover, when a pre-warming is combined to an intraoperative warming the extent of the drop in Tc during surgery and the incidence of hypothermia on awakening are reduced [27]. Our results confirm that at least 10 minutes of pre-warming combined with intraoperative warming for the entire time under anesthesia is effective and halves the incidence of hypothermia at arrival in RR.

Core temperature does not decrease evenly during the anesthesia. In our study, an initial phase represents roughly 70% of the total decrease and an initial drop above 0.5°C doubles the risk of having a Tc<36°C on admission to the RR. This result suggests that initial decrease in core temperature cannot be easily compensated for during surgery, regardless of the duration and the use of cutaneous warming. To avoid the initial drop in Tc, some have suggested warming the peripheral compartment before induction of anesthesia (pre-warming) [1]. Combining pre-warming and perioperative warming reduces the extent of the drop in Tc during surgery and the incidence of hypothermia on awakening [27]. Our results confirm that at least 10 minutes of pre-warming combined with intraoperative warming for the entire time under anesthesia is effective [21] and halves the incidence of hypothermia at arrival in RR.

In our study, one-third of the patients left the RR with a Tc<36°C. Absence of Tc monitoring in RR (62%) probably partially explains this. Interestingly, some patients became hypothermic during their stay in the RR, probably in relation with thermoregulatory effects of residual anesthetic medications or of analgesics [1,28]. Thus, the temperature must be measured several times during the stay to ensure that the patient leaving the RR is normothermic.

It has been reported that surgery lasting for more than 2 hours [9] represents a risk factor for hypothermia. In our study, the risk of hypothermia on admission to the RR is significantly influenced only by anesthesia lasting from 1 to 2 hours. However, if anesthesia lasted less than 1 hour or more than 2 hours, a large proportion of patients was hypothermic on awakening. Duration of the procedure should not be a factor determining the use of an active warming device except for anesthesia lasting less than 30 minutes.

In line with another report [29], we observed that 16.2% of the patients had a Tc<36°C before induction of anesthesia. A core temperature below 36°C could be considered as *normal* in a small part of the population [30]. However, we identified premedication with a benzodiazepine as a significant factor. A Tc<36°C before induction of anesthesia could result from the combination of exposition to cool temperatures in the holding area and the inhibitory effect of benzodiazepine on thermoregulation [31].

Temperature monitoring is recommended in order to motivate healthcare professionals to prevent hypothermia by identifying it [12,13]. Our results indicate that temperature monitoring is mainly performed by professionals who use active warming systems, without making any real impact on the incidence of hypothermia. Tc monitoring must therefore be encouraged, but it must also be supported by providing training about warming techniques.

This study has some limitations. A first limitation is that the instruction for the local investigator was to act “as usual”. This instruction has probably been influenced by the fact to participate to a study on the prevalence of perioperative hypothermia and by the presence of a CRA, and has potentially induced a bias. Of course, the presence of the CRA might have influenced the professional in his practice. However, more than 1 patient out 5 was not actively warmed or warmed inappropriately despite the presence of the CRA, suggesting that the behavior was not modified in a sense of good practice in many cases.

Our results suggest that modalities (pre- and/or intra-operative) and duration of warming are key points for avoiding hypothermia at the end of the surgery. However, one cannot on the basis of this study conclude that a pre-warming should be systematically associated to an intraoperative warming. Firstly, we conducted a pragmatic and not a randomized study comparing the two modalities. Then, even if a trained clinical research assistant collected data related to warming modalities, in particular when warming started and stopped, the periods when cutaneous warming was interrupted (i.e. to place the surgical drapes), were not recorded and, thus were not subtracted from the total duration of warming. Also, more evidence is needed to determine if the association of an active pre- and intraoperative warming is really more effective than an active intraoperative warming continued throughout the anesthesia. Another limitation is that all professionals working in the same health facility were considered as applying the same hypothermia prevention policy because it was impossible to conduct an exhaustive study across all the anesthesiologists. This is probably not entirely accurate. However, our results allowed us to establish that different types of healthcare facilities follow different perioperative active warming policies.

## Conclusion

Today a majority of surgical patients in France continue to be affected by post-anesthesia hypothermia. This is not because warming systems are under-utilized; it is because they are poorly utilized. In other words, “putting an active warming blanket on the patient” is not sufficient to prevent perioperative hypothermia. Moreover, our results suggest that only a combination of pre-warming and intraoperative warming throughout the anesthesia significantly reduces the incidence of hypothermia. Our results should encourage professionals to make the best possible use of active warming systems and monitor patient temperatures in order to better identify the prevalence of hypothermia.

## Supporting information

S1 Appendix Anonymized dataset. (XLSX)

S2 Appendix TREND checklist. (PDF)

S3 Appendix Protocol. (7Z)
